# Rising socioeconomic disparities in childhood overweight and obesity in Belgium

**DOI:** 10.1186/s13690-024-01328-y

**Published:** 2024-07-02

**Authors:** Sabine Drieskens, Rana Charafeddine, Stefanie Vandevijvere, Robby De Pauw, Stefaan Demarest

**Affiliations:** 1 Epidemiology and public health, Sciensano, J. Wytsmanstreet 14, Brussels, 1050 Belgium; 2 Department of Rehabilitation Sciences, Ghent University, Ghent, Belgium

## Abstract

**Background:**

Childhood overweight, especially obesity, significantly impacts children’s health and poses an increased risk of adult-onset diseases. This study aims to analyse the evolution of childhood overweight and obesity in Belgium from 1997 to 2018 and assess its variation across parental socioeconomic status (SES).

**Methods:**

The Health Interview Survey, a cross-sectional survey representative of the Belgian population, has been conducted since 1997, with the latest survey conducted in 2018. This study focuses on children aged 2–17 years. Body Mass Index (BMI, kg/m²) was derived from self-reported data, supplemented with proxy reports for children under 15 years old. Overweight and obesity were classified using age/sex-specific cut-off points. Highest parental educational level served as the indicator of SES. In addition to reporting the overall prevalence and the 95% confidence interval (95%CI) of childhood overweight and obesity by year, this study examines the absolute difference in prevalence between SES groups (low minus high) and calculates the Odds Ratio (OR, adjusted for age and sex) to evaluate the relative difference.

**Results:**

The overall prevalence of childhood overweight rose from 13.6% (95%CI = 11.2-16.1%) in 1997 to 18.9% (95%CI = 16.3-21.5%) in 2018; while it remained stable for obesity, fluctuating between 5.4% and 6.3% over the same period. This increase was more pronounced among children with low SES compared to those with high SES. Consequently, the absolute difference between children with low and high SES increased over time from 8.0% points (pp) in 1997 to 14.9 pp in 2018 for overweight, and from 3.1 pp to 6.8 pp for obesity. In terms of relative inequalities, overall, children with low SES exhibited significantly higher odds of overweight and of obesity than those with high SES (OR varying between 2 à 3 for overweight and between 2 and 4 for obesity).

**Conclusions:**

The escalating disparities over time highlight SES as a significant risk factor for childhood overweight and obesity. Addressing these inequalities requires interventions such as providing healthy meals and increasing sports opportunities at school. Additionally, it is recommended to regulate fast food outlets near schools and limit unhealthy food marketing, particularly because children with low SES are more exposed to such influences.

**Table:** 

Text box 1. Contribution to the literature
• Despite numerous studies examining trends in childhood overweight and obesity concerning socioeconomic status, little attention has been paid to the situation in Belgium.
• To assess socioeconomic inequalities effectively, the optimal approach involves relying on a set of inequality measures to capture various aspects. Here the metrics employed include absolute prevalence difference and odds ratio.
• Given the increasing trend of childhood overweight also in Belgium, with escalating socioeconomic disparities, proactive prevention such as promoting healthy food in school and creating healthy food environments near school are crucial.

## Background

Childhood overweight, especially obesity, has a major impact on children’s quality of life. In the short term, children with overweight have more metabolic and cardiovascular risk factors, such as type 2 diabetes and high blood pressure [1]. Additionally, overweight can trigger psychosocial problems among children and adolescents, including stigmatization, bullying, social exclusion, low self-esteem, and depression [1–7]. These issues can negatively impact school performance and may lead to lower educational attainment [3, 4, 8]. In the long term, excess weight in childhood strongly predicts overweight in adulthood, increasing the risk of chronic diseases, such as diabetes, cardiometabolic disease and certain cancers such as leukaemia, Hodgkin’s disease, colorectal cancer and breast cancer, consequently leading to premature mortality [1, 5–7]. Therefore childhood overweight and obesity particularly, is an important concern that should be addressed to reduce these risks [6], with children being a key target for early intervention [9].

In most cases, overweight is caused by a positive energy imbalance, where calorie intake through food and drinks exceeds calorie expenditure through physical activity [2, 4, 10]. Therefore, poor eating habits and a lack of physical activity are significant risk factors for weight gain. The consumption of free sugars, particularly in sugar-sweetened beverages, is strongly associated with overweight among children and adolescents [10, 11]. Moreover, young people are being less physically active due to a sedentary lifestyle, particularly as time spent on screen-based activities is increasing [10, 11]. These adverse behaviours are stimulated by an obesogenic environment where cheap, energy dense, nutrient-poor food is readily available and heavily marketed, opportunities for physical activity such as active transport are decreasing, and exposure to TV and computer devices is increasing [5, 6, 10, 12, 13]. A recent study of Smets et al. conducted in the Flemish part of Belgium indicated that the food environments around schools became unhealthier between 2008 and 2020. This means that children, who are highly susceptible, are increasingly exposed to unhealthy food retailers such as convenience stores, fast food outlets, takeaways and delivery services. The study also found an association between these food environments and overweight among young children (< 12 years) [14].

A pooled study of 2416 populations worldwide has shown that the increasing trend in the mean Body Mass Index (BMI) among children and adolescents (5–19 years) has been levelling off in many high-income countries since around 2000 [3]. The Childhood Obesity Surveillance Initiative (COSI), a study conducted in the World Health Organization (WHO) European Region among children aged 6–9 years, found a similar trend of stabilization in the Northern and Eastern European countries, and even a decline in the Southern European countries, regarding the prevalence of overweight and obesity from 2007 to 2017 [13]. Despite the overall prevalence of overweight and obesity stabilizing in recent years, several studies in European countries have shown that socioeconomic inequalities in overweight and obesity have increased, and this at the expense of children and adolescents from lower socioeconomic status (SES) backgrounds [5, 15–18]. Children from families with lower SES are more vulnerable to food insecurity and engage in less healthy dietary behaviours. They also tend to be less physically active [5, 10, 11, 19]. Studies have shown that unhealthy food environments are often located near schools in deprived neighbourhoods [10, 14, 20]. Additionally, deprived neighbourhoods generally have limited access to sport facilities and green spaces [10, 19].

Given the lack of studies on the evolution of childhood overweight and obesity over the past two decades in Belgium, this study aims to address two main objectives. Firstly, we aim to examine how the prevalence of overweight and obesity among children and adolescents (2–17 years) has evolved between 1997 and 2018 in Belgium. Secondly, we aim to explore whether this evolution differs based on parents’ SES, particularly their educational attainment [21].

## Methods

### Survey methodology [22]

Since 1997, Sciensano, the Belgian Institute for Health, has conducted six successive Health Interview Surveys (HIS) in Belgium, with additional surveys carried out in 2001, 2004, 2008, 2013 and 2018. The HIS target population compromises all residents of Belgium, utilizing quarterly updates of the National Population Registry (NPR) as the sampling frame. The HIS is a cross-sectional interview survey at household level. Respondents are selected using a multistage sampling design that includes geographical stratification, selection of clusters within each stratum, selection of households within each cluster, and selection of individuals within each household.

The total sample size of the HIS is 10,000 individuals and this sample is representative for Belgium as well as the main regions. Additionally, within both the Flemish and Walloon Regions, a second level of stratification is applied at the provincial level. In each stratum, groups of 50 individuals (12.5 by quarter) are selected from a limited number of municipalities. Within each group, households are selected via a systematic sampling procedure, based on statistical sector, household size and the age of the household’s reference person. A maximum of four members per household are invited for participation. In households with more than four members, the reference person and their partner are always selected, along with two or three other members of the household.

To ensure the target number of interviews is achieved on time, matched substitution is applied. For each selected household, seven consecutive households from the ranked list used in systematic sampling are designated as substitutes. The selected household and its substitutes are collectively referred to as a cluster. The initial selected household and its substitutes are similar in terms of statistical sector, household size and the age group of the reference person. If the first household in the cluster does not participate, the next household in the cluster will be contacted and this process continues until the cluster is exhausted. If the entire cluster is exhausted without securing participation, a substitute cluster is activated. However, the initial and substitute clusters do not share common characteristics regarding the age of the reference person, household size, or statistical sector. The response rate for the survey years ranged from 55 to 61%. The surveys were approved by the ethical committee of Ghent University Hospital (approval number for the last survey cycle 2017/1454). Participants in the HIS provided their consent by taking part in the survey.

### Interviewers

In addition to sample selection, the fieldwork for the HIS was managed in collaboration with Statbel, the Belgian statistical office, which specializes in conducting Face-to-Face (F-to-F) interviews. Statbel has a pool of experienced interviewers who also worked on the HIS. Interviewers were required to attend a one-day training session related to the HIS. The practical aspects of the training were provided by staff of Statbel, while the HIS-team from Sciensano covered the content of the questionnaires.

### Questionnaire

The HIS consists of a F-to-F questionnaire and a paper based self-administered questionnaire (for participants aged 15+). Until 2008, a Paper and Pencil Interview (PAPI) was applied for the F-to-F interview, after which a Computer Assisted Personal Interview (CAPI) was adopted. The variables used in this study were all included in the F-to-F questionnaire, and the questions pertaining to these variables have remained consistent over time. The data collected are self-reported.

### Childhood overweight and obesity

To assess childhood overweight and obesity, participants were asked the following questions about body weight and height: ‘How much do you weigh without clothes and shoes? (kg)’ and ‘How tall are you without shoes? (cm)’. For children under the age of 15, parents provided the responses. Based on this information, the BMI was calculated by dividing weight by the square of height (kg/m²). Extreme values considered as biologically implausible were excluded. Age- and sex-specific BMI cut-off points recommended by the International Obesity Task Force (IOFT) were then applied to classify individuals as (1) overweight (including obesity) and (2) obesity in the age group 2 to 17 years [23]. Hereafter, the term ‘overweight’ will refer to both overweight and obesity.

### Parental educational level

Research indicates that parental education shows the strongest association with overweight and obesity compared to parental occupation and income [5, 8, 24, 25]. Additionally, education plays an important role in improving occupational opportunities and income level [24, 26]. Consequently, we have selected the variable ‘highest educational level of the household’ as the indicator of SES, assuming it primarily pertains to the parents. This educational level is assigned to all household members. It is defined according to the International Standard Classification of Education (ISCED), which comprises the following categories: (1) Primary education, (2) Lower secondary education, (3) Upper secondary education, (4) Post-secondary non-tertiary education, (5) Short-cycle tertiary education, (6) Bachelor’s or equivalent level, (7) Master’s or equivalent level and (8) Doctoral or equivalent level [27]. Eurostat proposes three main aggregates to present educational levels: low (categories 1 and 2), intermediate (categories 3 and 4) and high (categories 5–8) [28]. However, to avoid small cell counts, we have dichotomized this variable into low (categories 1–4) and high (categories 5–8) educational levels.

### Study population

The sample comprised children aged 2 to 17 years with complete information on BMI and parental educational level. The covariate ‘sex’ was categorized into boys and girls, and the covariate ‘age’ was regrouped in four categories: 2–4 years, 5–9 years, 10–14 years and 15–17 years. These age categories allow for differentiation between toddlers/pre-schoolers, middle childhood, young teens, and teenagers. The total sample size was 10,084 individuals; the distribution by survey year was as follows: 1,584 for 1997, 1,845 for 2001, 1,643 for 2004, 1,477 for 2008 1,677 for 2013 and 1,858 for 2018.

### Statistical analysis

The trends in childhood overweight and obesity were assessed by calculating weighted prevalence rates (with their corresponding 95% confidence intervals (95%CI)) stratified by survey year, for the total sample and disaggregated by parental educational level, sex and age of the children. To assess inequalities, the optimal approach is to rely on a set of inequality measures rather than on a single one because different measures show different aspects of the association between health and social status. For this end, we estimated the following two measures:

- The prevalence difference, which indicates the absolute difference in the prevalence between low and the high parental education levels.
- The odds ratio (OR), which indicates the relative difference between low and high parental education levels.

Both measures, the prevalence difference and the OR, offer insights about the polarization between parental educational levels [32]. They complement each other, as relative inequality measures allow to monitor inequalities by focusing on improving the health of disadvantaged groups, while absolute inequality measures give information on the absolute rate of childhood overweight and obesity within each group, thus allowing to judge the overall burden on public health [29].

Logistic regression models, stratified by survey year, were used to assess the OR and their 95%CI. Overweight or obesity was the dependent variable and parental educational level was the independent variable (with high educational level as reference category). Children’s age group and sex served as covariates. Significant differences were determined based on the p-value (< 0.05). Stratified analyses were deemed justified as the interaction between education level ad survey year showed significance within the general model for overweight, however not for obesity. The SURVEYLOGISTIC procedure was used taking into account the complex survey design (clustering and stratification) and the survey weights for estimating national results. All statistical analyses were performed with SAS 9.4.

## Results

Table 1 shows the distribution, including numbers and weighted prevalence rates, of overweight and obesity among children aged 2 to 17 years disaggregated by parental educational level, covariates and survey year. The overall prevalence of childhood overweight was significantly higher in 2018 (18.9% (95%CI = 16.3-21.5%)) compared to 1997 (13.6% (95%CI = 11.2-16.1%)), but not compared to the intermediate years. Regarding obesity, the overall prevalence stayed stable over the survey years, varying between 4.5% and 6.3%.

**Table 1:** Distribution (numbers and weighted prevalence rates) of overweight, including obesity (OWOB), and obesity (OB) among children aged 2–17 years by parental educational level, the covariates and survey year, Belgian HIS 1997–2018

	1997			2001			2004			2008			2013			2018		
	N	OWOB	OB	N	OWOB	OB	N	OWOB	OB	N	OWOB	OB	N	OWOB	OB	N	OWOB	OB
Overall	1584	13.6	4.5	1845	16.2	4.9	1643	16.3	5.2	1477	17.2	4.7	1677	18.1	6.3	1858	18.9	5.6
Parental educ. level																		
Low	921	16.8	5.7	1104	19.2	5.9	898	17.7	5.4	747	22.3	7.6	820	27.1	10.5	762	27.8	9.7
High	663	8.8	2.6	741	11.9	3.4	745	14.8	4.8	730	12.7	2.1	857	10.5	2.9	1096	12.9	2.9
Sex																		
Boys	788	14.2	4.5	948	16.7	5.0	860	18.4	6.7	755	18.3	5.2	865	18.8	5.6	917	17.4	4.7
Girls	796	13.0	4.5	897	15.8	4.7	783	14.2	3.5	722	16.0	4.2	812	17.3	7.1	941	20.5	6.7
Age group																		
2–4 years	308	15.3	5.0	343	18.8	9.8	310	19.2	10.9	302	19.4	8.6	355	21.8	10.1	358	24.5	11.7
5–9 years	533	14.6	6.8	549	18.4	6.5	463	21.6	6.4	419	20.1	7.4	526	22.2	7.9	623	18.5	5.0
10–14 years	436	15.2	3.2	563	17.2	3.2	542	14.7	2.8	463	17.5	1.9	471	15.4	4.2	574	20.1	4.3
15–17 years	307	9.2	2.3	390	10.2	1.3	328	8.8	2.9	293	10.7	1.4	325	12.1	3.7	303	12.0	3.3

**Fig. 1:**
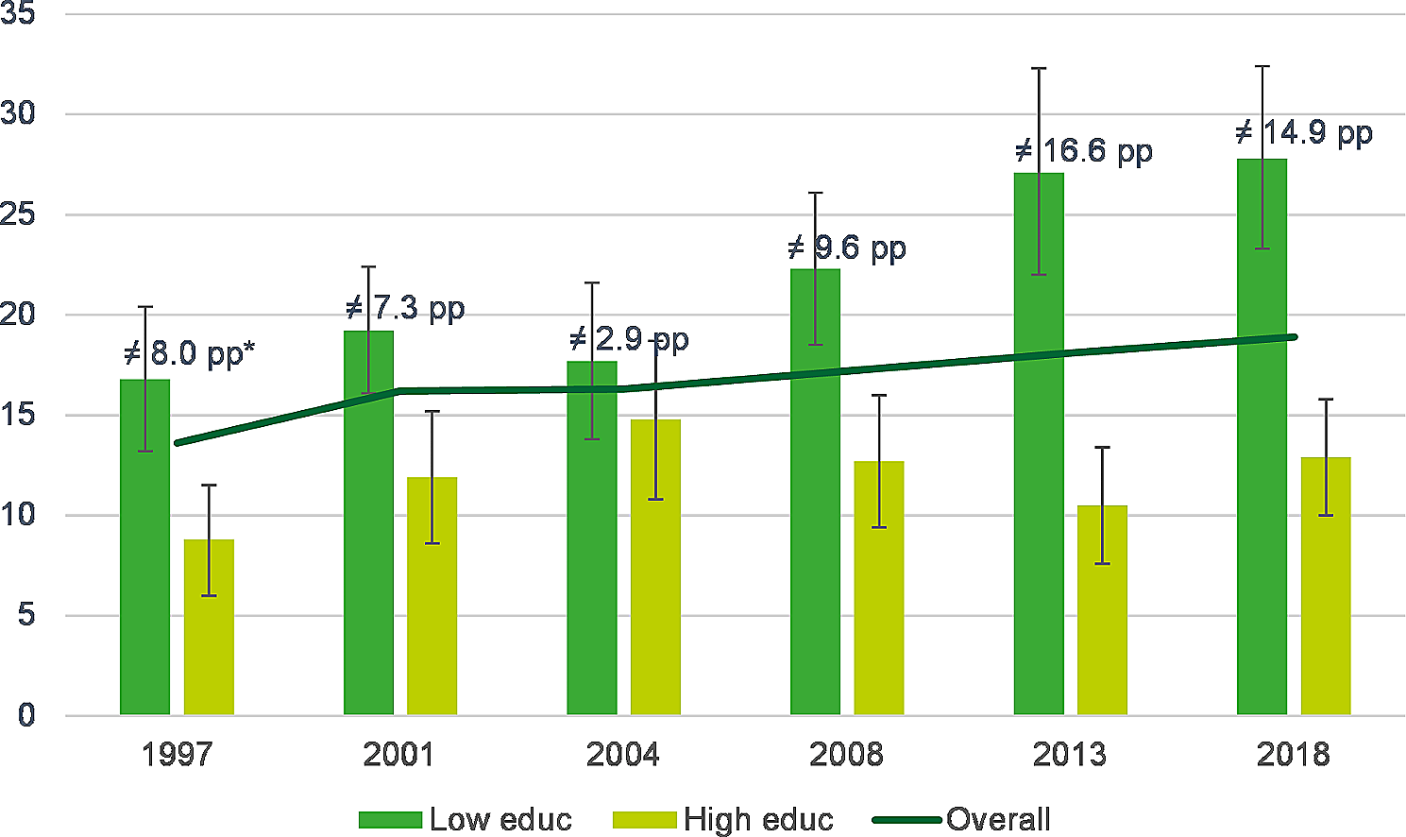
Evolution of the weighted prevalence rates of overweight, (including obesity), among children 2–17 years by parental educational level, Belgian HIS 1997–2018.* pp: percentage points

Figure 1 illustrates a substantial absolute difference, throughout the survey years, in childhood overweight between children of parents with a high educational level and those with a low educational level, with the exception of 2004. The evolution of the weighted prevalence of childhood overweight by parental educational level reveals a modest non-statistically significant rise in the prevalence of overweight among children of highly educated parents, increasing from 8.8% (95%CI = 6.0-11.5%) in 1997 to 12.9% (95%CI = 10.0-15.8%) in 2018. Contrastingly, children of parents with lower educational levels, exhibited a significant increase in overweight prevalence, climbing from 16.8% (95%CI = 13.2-20.4%) in 1997 to 27.8% (95%CI = 23.3-32.4%) in 2018. Consequently, the absolute difference between the low and the high educational groups widened over time, expanding from 8.0 pp in 1997 to 14.9 pp in 2018, with significant differences (non-overlapping 95% confidence intervals) observed for all survey years except 2004.

**Fig. 2:**
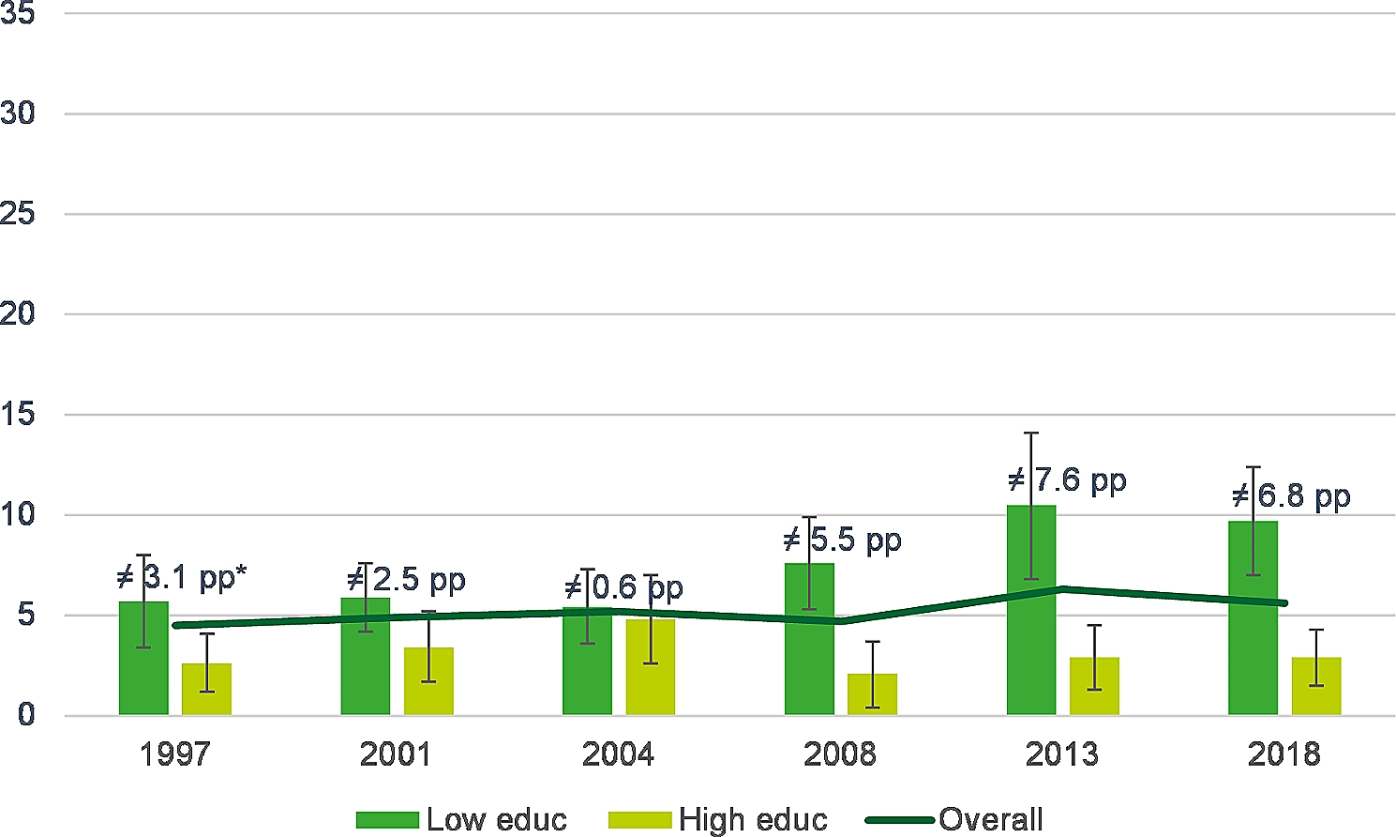
Evolution of the weighted prevalence rates of obesity among children 2–17 years by parental educational level, Belgian HIS 1997–2018.* pp: percentage points

Concerning obesity (Fig. 2), the weighted prevalence among children of highly educated parents remained relatively stable throughout the survey years, fluctuating between 2.1% and 4.8%. Conversely, for children of parents with lower educational levels, there appears to be a trend towards increased prevalence from 1997 to 2018, rising from 5.7% (95%CI = 3.4-8.0) to 9.7% (95%CI = 7.0-12.4%), although this trend lacked statistical significance. Nevertheless, the absolute difference between low and high educated levels increased from 3.1 pp in 1997 to 6.8 pp in 2018, with significant differences emerging 2008 onwards (non-overlapping 95% confidence intervals).

In Table 2, the ORs exhibit a consistent pattern corresponding to Fig. 1, indicating significantly higher odds of overweight among children of parents with lower educational levels compared to those with higher educational levels for all the survey years, except for 2004. Notably, the peak odds occurred in 2013 (OR = 3.39; 95%CI=[2.26–5.08]). Over time, these relative disparities remained persistent.

**Table 2:** Association between overweight, including obesity (OWOB) and obesity (OB) among youngsters and educational level of the parents, adjusted for sex and age, stratified by survey year, HIS 1997–2018

		OWOB		OB	
Survey year	Covariates	OR (95%CI)	P	OR (95%CI)	P
1997	Education (ref = high)	2.20 (1.41–3.44)	0.0005	2.52 (1.21–5.25)	0.0134
	Sex (ref = boys)	0.89 (0.59–1.34)	0.5742	1.01 (0.51-2.00)	0.9756
	Age (ref = 15–17 years)				
	2–4 years	1.92 (0.98–3.74)	0.0558	2.42 (0.79–7.44)	0.1231
	5–9 years	1.88 (1.02–3.46)	0.0429	3.56 (1.18–10.74)	0.0244
	10–14 years	1.81 (0.97–3.39)	0.0613	1.47 (0.46–4.68)	0.5171
2001	Education (ref = high)	1.89 (1.31–2.74)	0.0007	2.14 (1.13–4.05)	0.0197
	Sex (ref = boys)	0.92 (0.66–1.27)	0.5993	0.92 (0.56–1.53)	0.7569
	Age (ref = 15–17 years)				
	2–4 years	2.32 (1.37–4.02)	0.0028	9.40 (3.32–26.56)	< .0001
	5–9 years	2.16 (1.30–3.59)	0.0029	5.74 (2.08–15.87)	0.0008
	10–14 years	1.87 (1.14–3.06)	0.0130	2.57 (0.94–7.02)	0.0657
2004	Education (ref = high)	1.33 (0.88–2.01)	0.1743	1.29 (0.71–2.35)	0.4001
	Sex (ref = boys)	0.71 (0.50–1.01)	0.0589	0.48 (0.26–0.92)	0.0271
	Age (ref = 15–17 years)				
	2–4 years	2.56 (1.28–5.11)	0.0078	4.21 (1.43–12.41)	0.0091
	5–9 years	2.98 (1.64–5.40)	0.0003	2.41 (0.86–6.80)	0.0962
	10–14 years	1.79 (1.01–3.18)	0.0459	0.92 (0.30–2.82)	0.8915
2008	Education (ref = high)	2.03 (1.40–2.96)	0.0002	4.23 (1.76–10.21)	0.0013
	Sex (ref = boys)	0.85 (0.59–1.22)	0.3750	0.76 (0.40–1.45)	0.4043
	Age (ref = 15–17 years)				
	2–4 years	2.08 (1.17–3.71)	0.0131	7.40 (2.42–22.59)	0.0005
	5–9 years	2.22 (1.29–3.80)	0.0038	6.51 (2.17–19.54)	0.0009
	10–14 years	1.81 (1.02–3.19)	0.0408	1.42 (0.42–4.81)	0.5707
2013	Education (ref = high)	3.39 (2.26–5.08)	< 0.0001	4.28 (2.12–8.62)	< .0001
	Sex (ref = boys)	0.94 (0.65–1.36)	0.7523	1.38 (0.80–2.38)	0.2470
	Age (ref = 15–17 years)				
	2–4 years	2.36 (1.31–4.28)	0.0045	3.49 (1.44–8.42)	0.0055
	5–9 years	2.39 (1.39–4.10)	0.0017	2.60 (1.09–6.17)	0.0306
	10–14 years	1.43 (0.81–2.52)	0.2129	1.21 (0.42–3.50)	0.7232
2018	Education (ref = high)	2.66 (1.88–3.76)	< 0.0001	3.83 (2.17–6.76)	< .0001
	Sex (ref = boys)	1.28 (0.92–1.78)	0.1367	1.52 (0.96–2.39)	0.0733
	Age (ref = 15–17 years)				
	2–4 years	2.53 (1.36–4.69)	0.0034	4.25 (1.85–9.76)	0.0007
	5–9 years	1.72 (0.99–2.97)	0.0545	1.62 (0.74–3.51)	0.2244
	10–14 years	1.85 (1.07–3.18)	0.0271	1.32 (0.59–2.96)	0.5025

Concerning obesity, the odds were consistently higher among children of parents with lower educational levels compared to those with higher educational levels for all the survey years except 2004, which contrasts somewhat with the observed absolute differences, only reaching significance from 2008 onwards. However, starting from 2008, the odds were nearly double compared the preceding survey years.

## Discussion

In Belgium, the overall prevalence of childhood overweight saw a significant increase from 13.6% in 1997 to 18.9% in 2018, while obesity rates plateaued at 5.6% in 2018. The widening absolute socioeconomic disparities over time, from 8.0 pp in 1997 to 14.9 pp in 2018 for overweight, and from 3.1 pp to 6.8 pp for obesity, primarily stem from the escalating prevalence of overweight among children and adolescents of parents with lower educational levels, rising from 16.8% in 1997 to 27.8% in 2018 for overweight, and from 5.7 to 9.7% for obesity. Relative socioeconomic disparities remained relatively stable over time for both overweight and obesity.

The prevalence of childhood overweight in Belgium in 2018 (18.9%) can be contextualized by comparing it with similar studies conducted in other countries around the same time period. In the United States, the prevalence of overweight among children and adolescents aged 2–19 was notably higher at 35.4% in 2017–2018, as measured by weight and height [30]. In Slovenia, the prevalence stood at 24.4% among individuals aged 7–18 years in 2018 [12], while in Germany, it was reported as 15.4% among those aged 3–17 years during the period 2014–2017. Interestingly, both Slovenia and Germany exhibited stabilizing trends over time. In Slovenia, the prevalence of overweight rose from 13.7% in 1989 to 24.4% in 2018, but it appeared to stabilize after 2000 [12]. Similarly, in Germany, the prevalence stabilized around 2003–2006 at 15.5% [18].

It is worth noting that these studies were conducted prior to the COVID-19 pandemic. Recent research has highlighted a significant weight gain among children and adolescents during the COVID-19 lockdown, with those already overweight being at higher risk. The closure of (pre)school settings may have contributed to less healthy food consumption and reduced opportunities for structured physical activity [31–33].

The escalating socioeconomic disparities in childhood overweight in Belgium echo findings from other studies. A negative correlation between SES and overweight was also observed in France from 1999 to 2007 among children aged 3–14 years [25]. Similarly, in Germany spanning from 2003 to 2006 to 2014–2017 among children aged 7–18 years, a widening gap was noted: for low SES groups, overweight prevalence increased from 20.0% in 2003–2006 to 25.5% in 2014–2017, while it slightly decreased among middle and high SES groups [18].

Additionally, the Health Behaviour in School-aged Children (HBSC) survey, an international study tracking adolescents aged 11, 13 and 15 years every four years in the WHO European Region, revealed persistent socioeconomic disparities in overweight prevalence across most European countries from 2002 to 2014. In Belgium (Flemish part) and Iceland, however, these differences intensified due to a decline in overweight prevalence among adolescents from higher SES backgrounds and a simultaneous increase among those from lower SES background [10].

Frequently, healthy foods come with a higher price tag, while energy-dense options tend to be more affordable, exacerbating socioeconomic disparities in overweight. Consequently, policy interventions such as taxing sugar-sweetened beverages and subsidizing whole grains, fresh fruits, and vegetables are essential to make unhealthy food less accessible and promote healthier options, thereby addressing these inequalities [3, 5, 18]. Moreover, the widening socioeconomic gap in childhood overweight can be partly attributed to challenges in reaching and effectively communicating health information to parents with lower levels of education [15, 18, 19]. It is imperative to consider this communication problem with lower socioeconomic families should be taken into account when childhood overweight is addressed.

School-based interventions emerge as effective strategies to mitigate these disparities [4, 5, 18]. Schools should evolve into health-promoting environments [4], offering nutritious meals to increase fruit and vegetable consumption, and providing opportunities for physical activity [5, 6, 18]. Slovenia presents a compelling example of effective policy implementation within the educational system. Their initiatives focus on nutrition by eliminating foods with minimal nutritional value from school meals, emphasizing healthy dietary choices, promoting physical activity through an additional two hours of physical education per week. Moreover, they provide free-of-charge opportunities for physical activities within schools, particularly benefiting children who are not engaged in extracurricular sports [12].

Moreover, research indicates that children, particularly those in younger grades, are highly influenced by the food environments surrounding their schools [14, 20]. A study conducted in California unveiled an increase in fast food outlets near schools in deprived neighbourhoods, coupled with heightened exposure to advertisements promoting calorie-dense foods and beverages, potentially exacerbating socioeconomic disparities in childhood overweight [20]. Vandevijvere et al. have illustrated that individuals from lower socioeconomic backgrounds are disproportionately exposed to unhealthy food environments and marketing [34]. According to the Healthy Food Environment Policy Index (Food-EPI), national governments possess the greatest potential to enhance food and school environments [35]. Recommendations may include imposing limitations on the proliferation of fast food outlets and the availability of junk food and sugary beverages near schools, along with regulations restricting unhealthy food marketing [20, 35, 36].

This study possesses notable strengths, particularly its longitudinal data collection since 1997, employing consistent methodology and unchanged height and weight assessment questions. This continuity enables the determination of childhood overweight and obesity trends over the past two decades across a national representative sample. However, several weaknesses warrant acknowledge.

Firstly, the reliance on (parental) self-reported data constitutes a significant limitation. Studies have highlighted the inherent inaccuracies of self-reported height and weight, which could lead to BMI underestimation and consequently, an underestimation of overweight and obesity prevalence [37, 38]. Additionally, Lorant at all. observed a lower participation rate among low-educated individuals regarding health status when comparing the HIS 2001 with the Belgian Census 2001, possibly due to fear of stigmatization [39]. Despite these limitations, self-reported data collection in large population surveys remains more practical and cost-effective than measuring height and weight [37]. Another limitation pertains to the study’s singular focus on parental education as a measure of SES is multifaceted, and other dimensions such as parental occupation and income may also influence childhood overweight and obesity and warrant consideration in interventions [5, 8, 24, 40]. Lastly, comparing the study’s results with those of other investigations is challenging due to variations in overweight and obesity classification systems, measurement methods (self-reported versus measured), and age group definitions [3, 7]. These discrepancies hinder direct comparisons and limit the broader contextualization of findings.

## Conclusion

In Belgium, the overall prevalence of childhood overweight saw a significant increase from 1997 to 2018, although the same trend was not observed for obesity. This rise was notable children of parents with a lower socioeconomic status, leading to a widening of absolute socioeconomic disparities over time, including for childhood obesity. However, the relative inequalities, as indicated by the odds ratio, did not exhibit a significant increase. Given the considerable public health implications, urgent policy interventions are required to prevent overweight and obesity, especially among children and adolescents from disadvantaged backgrounds. In addition to implementing initiatives within schools to promote and provide healthy food options and opportunities for physical activity, creating a healthy food environment around schools and imposing restrictions on unhealthy food marketing are essential. To effectively monitor the impact of these policies, continuous monitoring of childhood overweight and obesity through health surveys is necessary.

## Data Availability

The microdata of the HIS are available upon request. Detailed instructions for requesting access to microdata can be found on the official website: https://www.sciensano.be/en/node/55737/health-interview-survey-microdata-request-procedure.

## Abbreviations

NPR: National Population Registry
SES: Socioeconomic status
BMI: Body Mass Index
95%CI: 95% confidence interval
OR: Odds ratio
WHO: World Health Organization
HIS: Health Interview Survey
F-to-F: Face-to-Face
PAPI: Paper and Pencil Interview
CAPI: Computer Assisted Personal Interview
IOFT: International Obesity Task Force
ISCED: International Standard Classification of Education
pp: Percentage points
HBSC: Health Behaviour in School-aged Children
Food-EPI: Healthy Food Environment Policy Index
